# Complete Genome Sequence of Rift Valley Fever Virus Strain Lunyo

**DOI:** 10.1128/genomeA.00170-16

**Published:** 2016-04-14

**Authors:** Sarah Lumley, Daniel L. Horton, Denise A. Marston, Nicholas Johnson, Richard J. Ellis, Anthony R. Fooks, Roger Hewson

**Affiliations:** a Virology and Pathogenesis Group, Microbiology Services Division, Public Health England, Wiltshire, United Kingdom; b Department of Pathology and Infectious Disease, School of Veterinary Medicine, University of Surrey, Guildford, United Kingdom; c Wildlife Zoonoses and Vector-Borne Diseases Research Group, Animal and Plant Health Agency, Addlestone, Surrey, United Kingdom; d Specialist Scientific Support Department, Animal and Plant Health Agency, Addlestone, Surrey, United Kingdom; e Department of Clinical Infection, Microbiology and Immunology, Institute of Infection and Global Health, University of Liverpool, Liverpool, United Kingdom; f National Institute for Health Research, Health Protection Research Unit in Emerging and Zoonotic Infections, University of Liverpool, Liverpool, United Kingdom

## Abstract

Using next-generation sequencing technologies, the first complete genome sequence of Rift Valley fever virus strain Lunyo is reported here. Originally reported as an attenuated antigenic variant strain from Uganda, genomic sequence analysis shows that Lunyo clusters together with other Ugandan isolates.

## GENOME ANNOUNCEMENT

Rift Valley fever virus (RVFV) is a *Phlebovirus*, in the family *Bunyaviridae*, and was first isolated in 1930. It primarily affects ruminants, causing abortions and mortality, and in humans it causes a febrile illness, which is severe in 1% of cases (1). The virus cycles between its mosquito vector and mammalian hosts. The strain Lunyo was isolated from a pool of *Aedes* mosquitoes in the Lunyo Forest, Uganda, in 1955 (2). It was described as a variant strain due to varying reports of hemagglutinin and antibody neutralization (2–5). Pathogenicity in mice was initially low but increased with serial passage (2, 3), except in some strains of mice, hamsters, gerbils, and rats, where the virus remains attenuated (5, 6). Genome characterization is essential to improve understanding of the role sequence variation has on virulence.

Lunyo mouse brain suspension (*P* ≥ 11) was passaged once in Vero E6 cells. Viral RNA was extracted from supernatant using the QIAamp viral RNA minikit (Qiagen). Double-stranded (ds) cDNA was synthesized from 50 ng of RNA using the cDNA Synthesis System (Roche) in accordance with the manufacturer’s instructions. ds-cDNA was purified using Agencourt AMPure XP system (Beckman Coulter). A sequencing library was prepared from 1 ng of DNA using the Nextera XT-DNA library preparation kit (Illumina) according to the manufacturer’s instructions. Sequencing was performed following standard Illumina protocols on the Illumina MiSeq with 150-bp paired-end reads. Total reads (8,556,470) were mapped to reference sequences (EU312121, DQ380217, DQ375419) using BWA version 0.7.5a-r405 (7) and visualized in Tablet (8). Consensus sequence was generated in modified samtools/vcfutils (9), and intermediate sequence was used as a reference for subsequent iteration mapping (10). A total of 67,991 reads were mapped (0.79%), resulting in near-complete small (S) (18,889 reads), complete medium (M) (22,833), and large (L) (26,269) segments with an average read depth of 1,208, 623, and 443, respectively. The S segment intergenic region nucleotides were resolved by amplicon-based next-generation sequencing using primers binding to terminal regions (11).

Phylogenetic analyses of the S, M, and L segments were performed in MEGA6. All complete RVFV sequences in GenBank (*n* = 160, 99, and 94 for the S, M and L segments, respectively) were aligned by ClustalW, and a maximum likelihood phylogenetic tree was constructed for each genome segment. Analysis of novel sequence data showed that all three Lunyo segments cluster with strains originating from Uganda, within the previously defined lineage E (12). Genetic similarity to a closely related strain isolated from mosquitoes in Entebbe, Uganda, in 1944 (DQ380156, DQ380191, DQ375429) demonstrated 97.5%, 98.6%, and 98.3% nucleotide identities in the S, M, and L segments, respectively. Results show 4 nonsynonymous substitutions in the nucleoprotein, 3 in the nonstructural protein, 8 in the glycoproteins, and 8 in the L protein. A single-nucleotide insertion in the intergenic region of the S segment conserves the segment size range of 1,690 to 1,962 (12). The contribution of a further complete genome to RVFV investigations will improve understanding of viral evolution and host interactions.

### Nucleotide sequence accession numbers.

Complete genomic sequences of RVFV strain Lunyo S, M, and L segments have been deposited in GenBank under accession numbers KU167025 to KU167027.
